# Evaluating the biopsy-negative prostate lesions based on sequential biparametric prostate magnetic resonance imaging: Can PRECISE be used to guide repeat biopsy?

**DOI:** 10.1186/s13244-026-02373-7

**Published:** 2026-09-07

**Authors:** Yuxuan Tian, Mingjian Ruan, Yuke Chen, Qi Shen, Shuoyan Chen, Jingyun Wu, Derun Li, Yu Fan, Yi Liu

**Affiliations:** 1 Department of Urology, Peking University First Hospital, Beijing, China; 2 Institute of Urology, Peking University, Beijing, China; 3 National Urological Cancer Center of China, Beijing, China; 4 Department of Radiology, Peking University First Hospital, Beijing, China; 5 Drug Clinical Trial Institution, Peking University First Hospital, Beijing, China

**Keywords:** Magnetic resonance imaging, Biopsy, Prostatic neoplasms

## Abstract

**Objective:**

Indications for repeat biopsy after an initial negative prostate biopsy remain poorly defined. Therefore, this study aimed to investigate the effectiveness of the Prostate Cancer Radiological Estimation of Change in Sequential Evaluation (PRECISE) scoring system as a guide for repeat biopsy.

**Materials and methods:**

We retrospectively reviewed patients who underwent repeat prostate biopsy and had bpMRI performed before both biopsies between 2013 and 2024. The association of clinically significant prostate cancer (csPCa) detection with initial Prostate Imaging Reporting and Data System (PI-RADS) and PRECISE scores was analyzed. The predictive effects of prostate-specific antigen density before repeat biopsy (re-PSAD) and PSA velocity (PSAV) on csPCa were assessed in patients progressing to or maintaining re-PI-RADS 3.

**Results:**

104 individuals were included with a median follow-up period of 28.58 months and a PSAV of 0.90 ng/mL/year (interquartile range (IQR), −0.17 to 2.57 ng/mL/year). The csPCa detection rate at repeat biopsy was 24.0%. 42 participants (40.4%) had PRECISE scores of 4–5, 43 (41.3%) had scores of 3, and 19 (18.3%) had scores of 1–2. A higher PRECISE score corresponds to a higher csPCa detection rate, with csPCa diagnosed in 52.4% of patients with PRECISE scores of 4–5. In subgroup analyses, re-PSAD and PSAV avoided 65.4% and 53.8% of repeat biopsies, respectively, and would have caused 5.9% and 0.00% of missed csPCa.

**Conclusions:**

PRECISE may be useful for guiding repeat biopsy decisions and may provide incremental value for predicting csPCa when interpreted together with PI-RADS. Additionally, PSAD and PSAV can assist in screening for repeat biopsy.

**Key Points:**

- ***Question*** Indications for repeat prostate biopsy after an initial negative result remain poorly defined. Can sequential MRI PRECISE scoring help inform repeat biopsy decisions?
- ***Findings*** In our retrospective cohort, PRECISE 1–2 yielded no clinically significant cancer, while PRECISE 4–5 showed a 52.4% detection rate. PSA parameters aided equivocal cases.
- ***Critical relevance statement*** This study evaluates sequential MRI PRECISE scoring, suggesting its potential to help reduce unnecessary repeat biopsies for regressing lesions while identifying high-risk progressing abnormalities.

**Graphical Abstract:**

**Figure:**
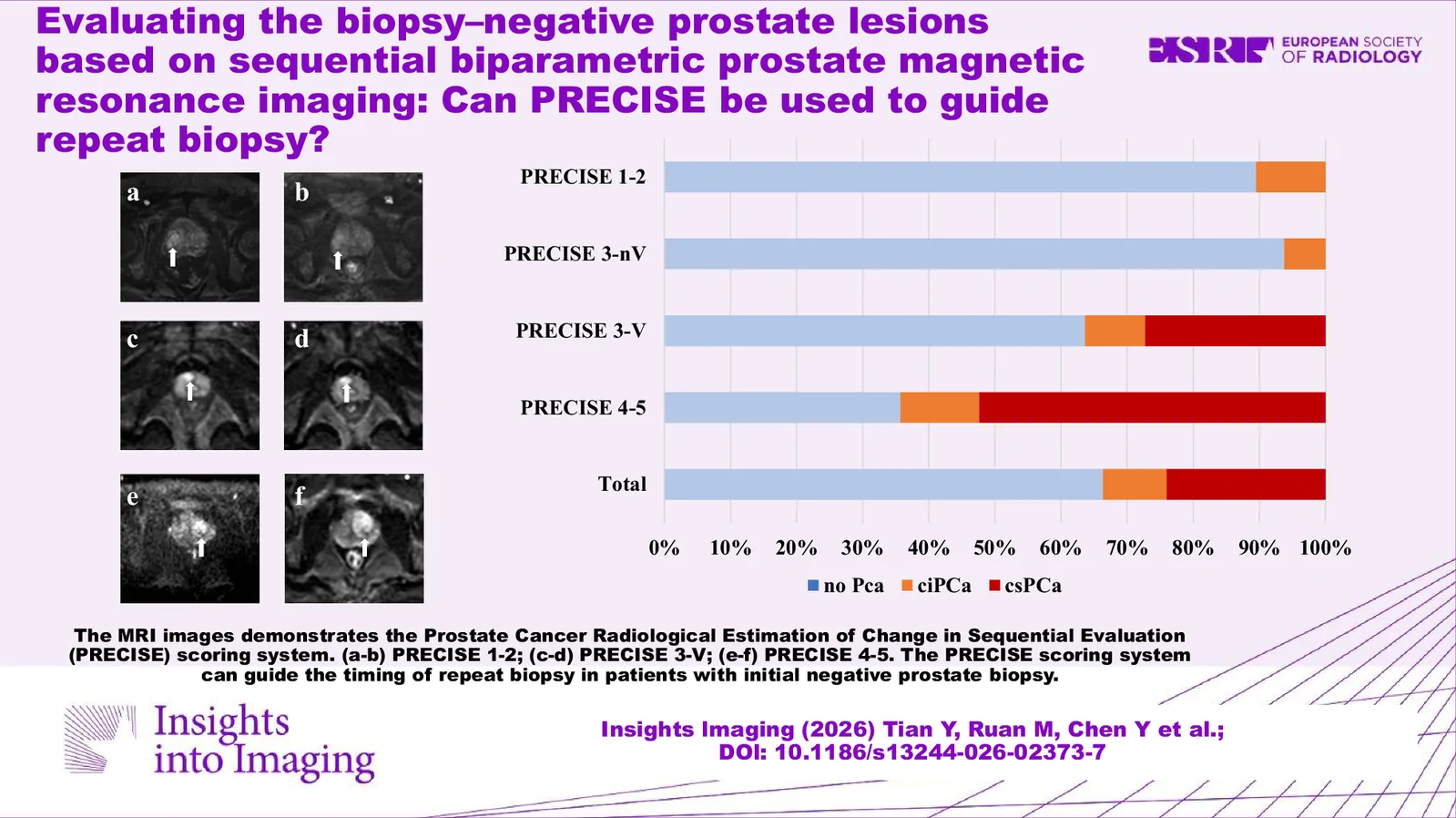

## Introduction

Prostate biopsy remains the gold standard for the diagnosis of prostate cancer (PCa) [1]. Nevertheless, negative results from either systematic or targeted biopsies do not entirely rule out prostate cancer [2, 3], and indications for repeat biopsy in this setting are not yet standardized. Multiparametric magnetic resonance imaging (mpMRI) has demonstrated high sensitivity for detecting clinically significant prostate cancer (csPCa) [4, 5]. Recent evidence suggests that biparametric MRI (bpMRI) offers comparable diagnostic accuracy while improving cost-effectiveness and accessibility [6–8]. Serial MRI examinations are utilized during active surveillance follow-up to evaluate changes in prostate lesions [9, 10]; furthermore, follow-up strategies for patients with suspected prostate cancer have preliminarily demonstrated oncological safety [11].

While the Prostate Imaging Reporting and Data System (PI-RADS) is widely used for csPCa risk stratification [12, 13], it is not designed to assess radiological change over time. The Prostate Cancer Radiological Estimation of Change in Sequential Evaluation (PRECISE) recommendations provide a standardized method to assess tumor progression during active surveillance and have been applied in clinical practice and research [14–16]. PRECISE serves as a strong predictor of histological upgrading [17, 18]. Notably, previous studies have shown that, using a category ≥ 4 cutoff, the PRECISE scoring system predicts progression on active surveillance with a sensitivity of 0.76 and a specificity of 0.89 [19]. However, it remains unclear whether PRECISE can be applied to serial MRI in men with persistent clinical suspicion after a negative biopsy and whether it can guide decisions regarding repeat biopsy.

Therefore, this study aimed to evaluate the diagnostic value of PRECISE for detecting csPCa at repeat biopsy in patients with a prior negative biopsy. We also assessed clinical factors associated with cancer detection at repeat biopsy to improve risk stratification.

## Materials and methods

### Study population

**Fig. 1:**
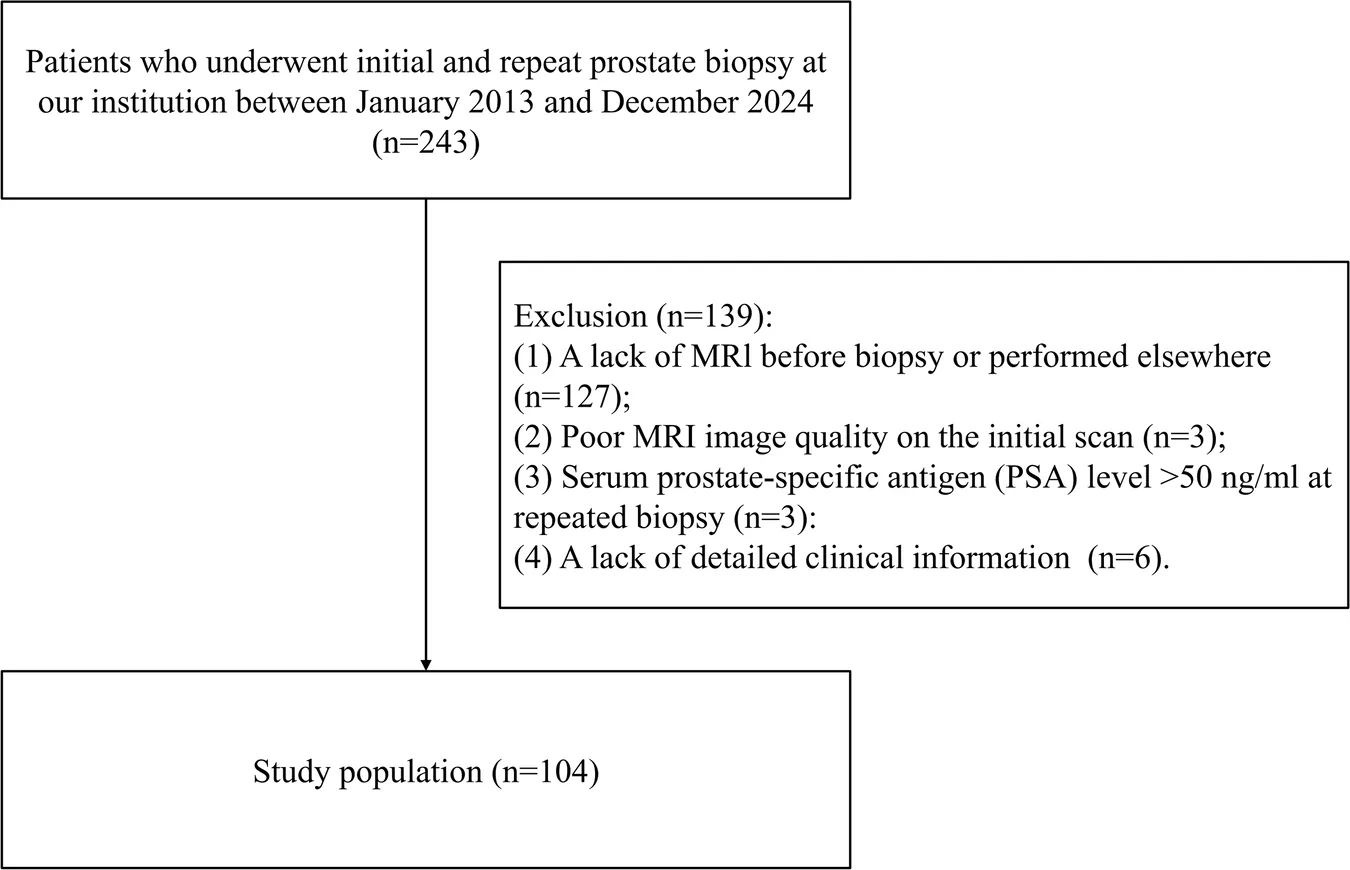
Flowchart of patient selection

This study was approved by the Ethics Committee of Peking University First Hospital (No. 2023YAN272-002). We retrospectively screened all consecutive patients (*n* = 243) who underwent prostate biopsy at Peking University First Hospital between January 1, 2013, and December 31, 2024. Overall, 139 patients were excluded based on the following criteria: (1) absence of bpMRI prior to biopsy or performed at other institutions (*n* = 127); (2) the initial MRI images were of poor quality (*n* = 3); (3) total prostate-specific antigen (tPSA) level exceeding 50 ng/mL (*n* = 3) and (4) lack of detailed clinical information (*n* = 6). Ultimately, 104 individuals were included in this study (Fig. 1).

### Magnetic resonance imaging

Prostate MRI was performed at our institution using pelvic phased-array coils (no endorectal coil). Scans acquired between 2013 and 2015 were performed on 1.5-T systems (HD and Achieva). From 2016 to 2024, examinations were performed on 3.0-T systems, including Discovery MR750 (GE Healthcare), Achieva (Philips Healthcare), and Signa EXCITE HD (GE Healthcare). The MRI protocol consisted of axial T2-weighted fast/rapid spin-echo images targeted to the prostate, axial T1-weighted imaging of the pelvis, and axial diffusion-weighted imaging (DWI) with b-values of 0, 800, and 1400 s/mm², with corresponding apparent diffusion coefficient (ADC) maps.

### Image analysis

MRI scans were retrospectively and independently evaluated by two experienced radiologists. Both readers were blinded to the pathological outcomes of the repeat biopsies. Using the PI-RADSv2.1 structured scoring criteria, the likelihood of a tumor was assessed [12]. Serial bpMRI scans were compared with the initial images by the two radiologists in accordance with the PRECISEv2 criteria [16]. In cases of scoring discrepancies between the two initial readers, a third senior consultant adjudicated the results to reach a consensus.

**Fig. 2:**
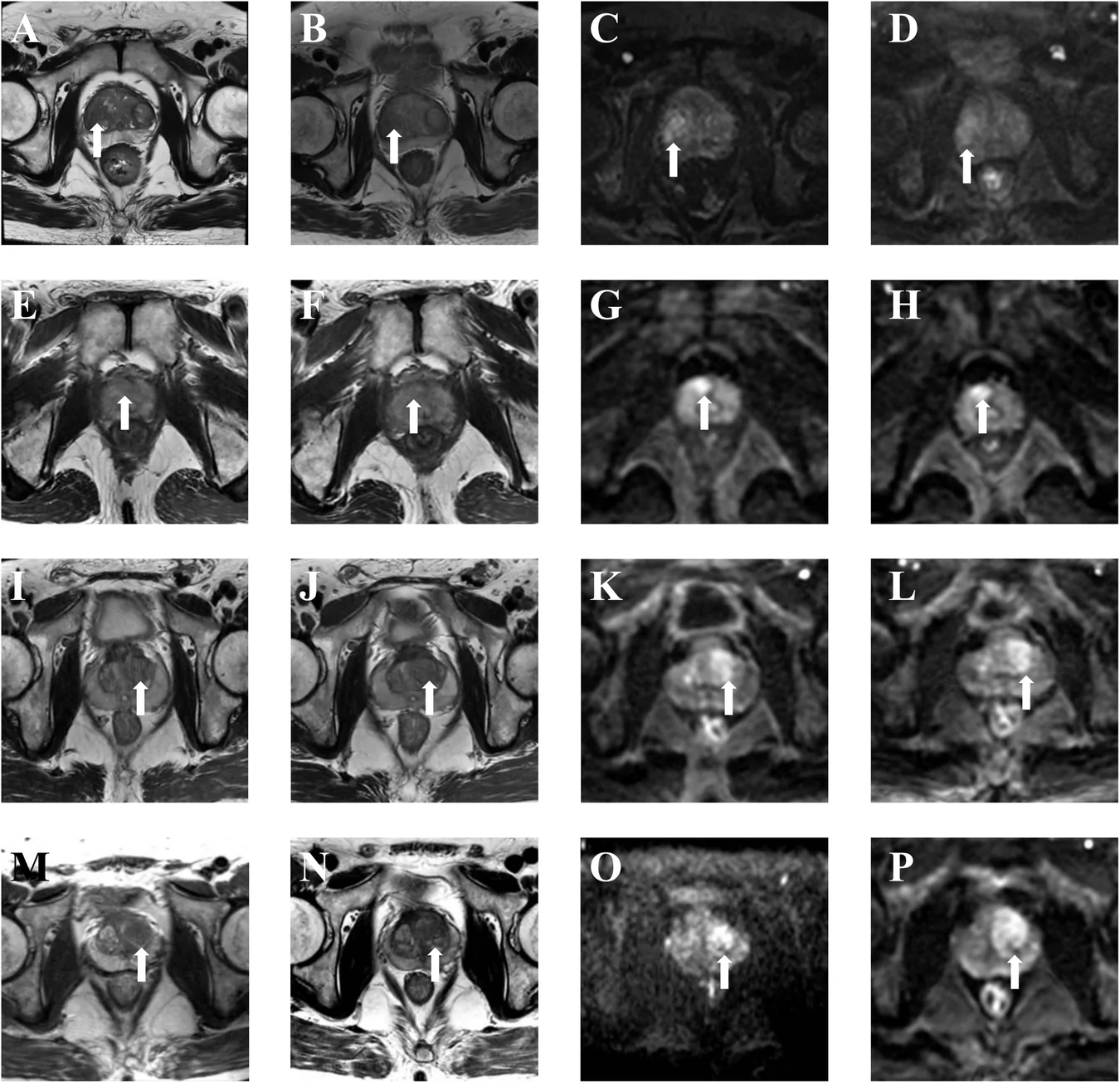
Representative examples of sequential biparametric MRI (bpMRI) assessments and repeat biopsy outcomes categorized by the PRECISE scoring system. All suspected lesions are indicated by arrows. **A**–**D** Radiological regression (PRECISE 2). A 65-year-old man with an initial negative prostate biopsy. Baseline MRI (**A**, **C**) shows a 2.3-cm PI-RADS 5 lesion in the right transition zone, which is hypointense on axial T2-weighted imaging (T2WI) (**A**) and hyperintense on diffusion-weighted imaging (DWI) (**C**). Follow-up MRI at 14 months (**B**, **D**) demonstrates a clear reduction in lesion size to 1.6 cm on corresponding T2WI (**B**) and DWI (**D**). The lesion was assigned a PRECISE score of 2. Repeat biopsy revealed no evidence of prostate cancer. **E**–**H** Radiological stability (PRECISE 3) with clinically significant prostate cancer (csPCa). Sequential MRIs demonstrate a persistently visible 0.8-cm focal lesion in the peripheral zone. The lesion remained unchanged and was scored as PI-RADS 3 on both the baseline (**E**, **G**) and follow-up (**F**, **H**) scans, yielding a PRECISE score of 3. Repeat targeted biopsy detected csPCa with a Gleason score of 3 + 4. **I**–**L** Radiological stability (PRECISE 3) without prostate cancer. Sequential MRIs show a persistent lesion in the transition zone assigned a PI-RADS score of 4 on both baseline (**I**, **K**) and follow-up (**J**, **L**) examinations. Because of the lack of significant radiological change, it was assigned a PRECISE score of 3. Repeat biopsy was negative for prostate cancer. **M**–**P** Radiological progression (PRECISE 4). A 57-year-old man whose initial biopsy indicated only inflammation. Baseline MRI (**M**, **O**) reveals a 1.4-cm PI-RADS 3 lesion in the left transition zone, appearing hypointense on T2WI (**M**) and hyperintense on DWI (**O**). Follow-up MRI at 22 months (**N**, **P**) demonstrates increased focal conspicuity and signal intensity on corresponding T2WI (**N**) and DWI (**P**). Both radiologists assigned a PRECISE score of 4. Subsequent targeted biopsy confirmed csPCa with a Gleason score of 4 + 3

Our assessment of the MRI images, referring to the PRECISE criteria, is illustrated in Fig. 2. The PRECISE guidelines offer a five-point likelihood scale to ascertain radiological progression on MRI through serial prostate bpMRIs, which is primarily employed in patients undergoing active surveillance. A PRECISE score of 1–2 indicates resolution or regression of previously suspicious MRI features, based on a reduction in radiological size, stage, conspicuity, or PI-RADS score. Figure 2A–D illustrates these imaging changes, with the final pathological findings being consistent with inflammation and no evidence of csPCa. A score of 3 suggests radiological stability, whereas a score of 4–5 indicates disease progression on MRI, as evidenced by an increase in radiological size, stage, or conspicuity. These changes are illustrated in Fig. 2. In addition, the PRECISE 3 can also be classified into 3 visible (3-V) and 3 nonvisible (3-NonV) based on whether there is a visible focal lesion.

### Biopsy and pathology

All patients underwent transrectal systematic biopsies, consisting of 12 to 13 samples, and/or two or more MRI/ultrasound fusion-targeted biopsies, all performed under local anesthesia. To ascertain the Gleason score in cases where cancer was detected, each biopsy sample was placed in individual containers and analyzed by two specialized genitourinary pathologists. Prostate cancers with a Gleason score of 3 + 3 were categorized as clinically insignificant prostate cancer (ciPCa), irrespective of the length of the cancer core. Conversely, prostate cancers with a Gleason score of 3 + 4 or higher were classified as clinically significant prostate cancer (csPCa).

### Statistical analysis

Continuous variables were assessed for normality and reported as mean ± standard deviation (SD) or median (interquartile range [IQR]), as appropriate. Categorical variables are presented as frequencies and percentages. Inter-reader agreement for PRECISE scoring was assessed using Cohen’s kappa (κ) for both the 5-category scale (1–5) and a 3-category grouping (regression: 1–2; stability: 3; progression: 4–5). The association between initial PI-RADSv2.1/PRECISE scores and pathological outcomes was analyzed. Diagnostic performance of PSAV and re-PSAD was assessed using the receiver operating characteristic (ROC) curve analysis. The area under the curve (AUC), sensitivity, and specificity with 95% confidence intervals (CIs) were calculated, and optimal cutoff values were determined using Youden’s index. ROC analysis was performed using R software (v4.2.3; packages ‘pROC’ and ‘ggplot2’), while all other analyses were conducted using SPSS Statistics 26.

## Results

### PRECISE score agreement

In summary, the PRECISE score demonstrated substantial inter-reader agreement (κ = 0.74). When categorized into regression (PRECISE 1 and 2), stability (PRECISE 3), and progression (PRECISE 4–5), the agreement further improved (κ = 0.86).

### Study population characteristics

**Fig. 3:**
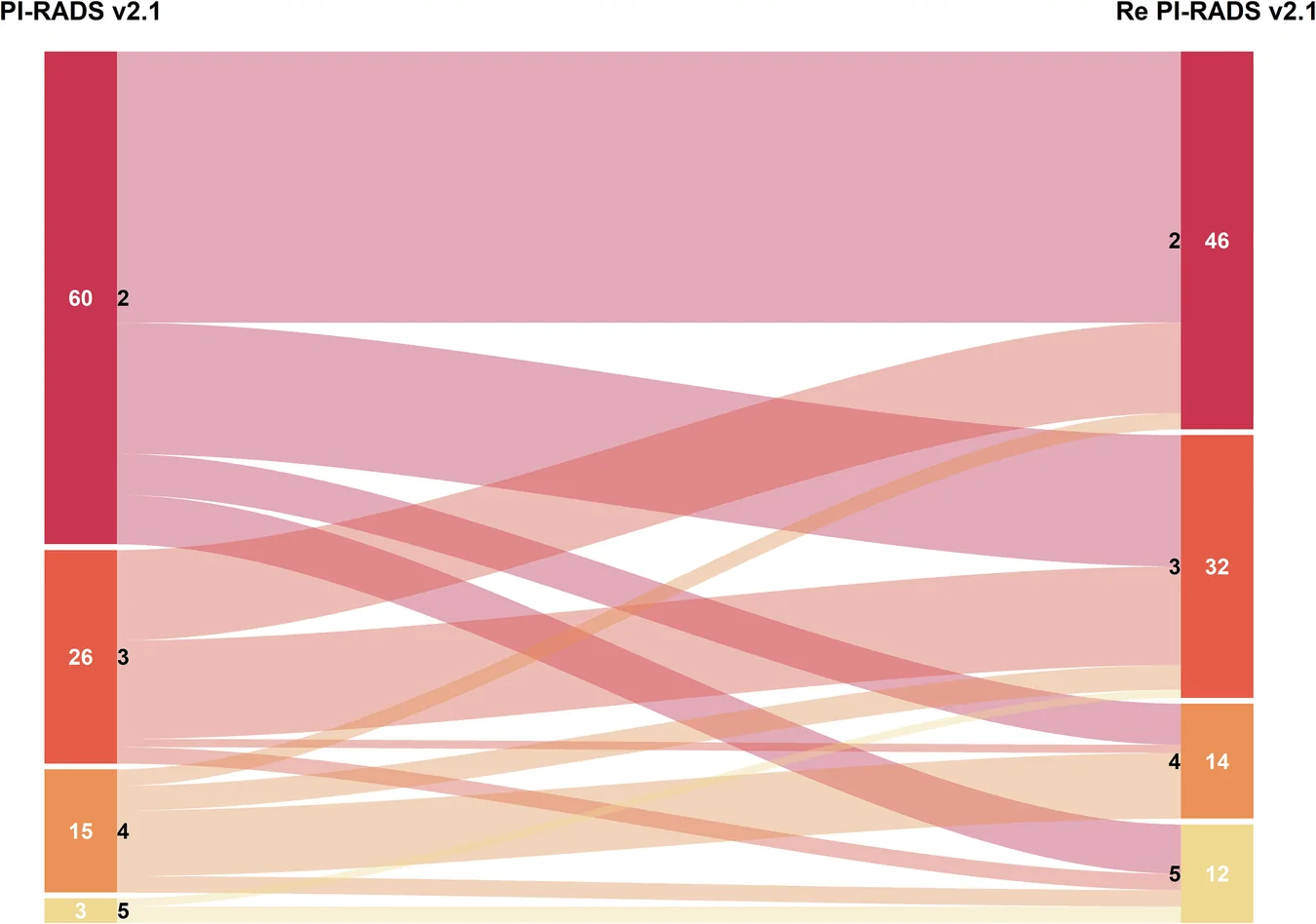
Sankey diagram depicting changes in PI-RADS in two MRI examinations of the same patient. PI-RADSv2.1, Prostate Imaging Reporting and Data System version 2.1; Re-PI-RADSv2.1, Prostate Imaging Reporting and Data System version 2.1 score on repeat MRI

Table 1 presents the clinical and pathological characteristics of the patients included in this study. The median tPSA levels were 8.98 (IQR, 7.60–14.17) ng/mL and 12.72 (IQR, 8.56–18.95) ng/mL for the initial and repeat biopsies, respectively. The mean age of the patients was 63.16 years (standard deviation, 6.47 years), and the median follow-up time was 28.58 months (IQR, 14.28–57.97 months), with a PSA velocity (PSAV) of 0.90 ng/mL/year (IQR, −0.17 to 2.57 ng/mL/year). Among the 104 patients, 42 (40.4%) exhibited radiographic progression (PRECISE 4–5) of their lesions, 43 (41.3%) had stable lesions (PRECISE 3), and 19 (18.3%) showed regression of their lesions (PRECISE 1–2). At initial biopsy, approximately half of the patients had pathological findings consistent with inflammation, while 2 (1.9%) showed high-grade prostatic intraepithelial neoplasia (HGPIN), and 23 (22.1%) had atypical small acinar proliferation (ASAP). After a repeat biopsy, prostate cancer was detected in 35 (33.7%) patients, of whom 25 (24.0%) had csPCa and 10 (9.6%) had ciPCa. Figure 3 illustrates changes in PI-RADS category between baseline and repeat MRI for each patient.

**Table 1:** Clinicopathological characteristics of the patients (*N* = 104)

Variable	
Age(y), Mean (standard deviation)	63.16 (6.47)
tPSA (ng/mL), Median (IQR)	8.98 (7.60–14.17)
PV (mL), Median (IQR)	58.95 (46.00–78.08)
PSAD (ng/mL^2^), Median (IQR)	0.16 (0.12–0.25)
Abnormal DRE, *n* (%)	7 (6.7)
Abnormal TRUS, *n* (%)	25 (24.0)
PI-RADSv2.1, *n* (%)	
2	60 (57.7)
3	26 (25.0)
4	15 (14.4)
5	3 (2.9)
Re-tPSA (ng/mL), Median (IQR)	12.72 (8.56–18.95)
Re-PV (mL), Median (IQR)	68.49 (48.95–93.19)
Re-PSAD (ng/mL^2^), Median (IQR)	0.19 (0.12–0.31)
Re-abnormal DRE, *n* (%)	13 (12.5)
Re-abnormal TRUS, *n* (%)	29 (27.9)
Re-PI-RADSv2.1, *n* (%)	
2	46 (44.2)
3	32 (30.8)
4	14 (13.5)
5	12 (11.5)
PRECISEv2.0, *n* (%)	
1–2	19 (18.3)
3-nonV	32 (30.8)
3-V	11 (10.6)
4–5	42 (40.4)
Follow-up time, (m), Median (IQR)	28.58 (14.28–57.97)
PSAV (ng/mL/y), Median (IQR)	0.90 (−0.17–2.57)
Initial pathological finding, *n* (%)	
Benign prostatic tissue	27 (26.0)
Inflammation	52 (50.0)
HGPIN	2 (1.9)
ASAP	23 (22.1)
Repeat pathological finding, *n* (%)	
PCa	35 (33.7)
csPCa	25 (24.0)
ciPCa	10 (9.6)

*ciPCa* clinically insignificant prostate cancer, *csPCa* clinically significant prostate cancer, *tPSA* total prostate specific antigen, *IQR* interquartile range, *PV* prostate volume, *PSAD* prostate-specific antigen density, *DRE* digital rectal examination, *TRUS* transrectal ultrasonography, *PI-RADSv2.1* Prostate Imaging Reporting And Data System version 2.1, *Re* repeat biopsy, *PRECISEv2.0* Prostate Cancer Radiological Estimation of Change in Sequential Evaluation version 2.0, *PSAV* prostate specific antigen velocity, *HGPIN* high-grade prostatic intraepithelial neoplasia, *ASAP* atypical small acinar proliferation.

### Sequential MRI and repeat biopsy pathology findings

In order to ascertain the optimal timing for repeat biopsies in patients who initially present with negative results, we conducted an analysis incorporating the initial PI-RADS score in conjunction with the PRECISE score, which captures the dynamic changes in lesions over time (Table 2). Among the men showing radiological regression (PRECISE 1–2), no csPCa was identified (0/19), regardless of their initial PI-RADS score. With a PRECISE score of 4–5, there was a 35.7% csPCa detection rate in initial biopsy-negative patients with an initial PI-RADS score of 2, and this detection rate reached 100% for patients with an initial PI-RADS score of 4–5. For patients with an initial PI-RADS score of 2, only two cases (6.2%) of ciPCa were detected when the imaging lesion remained stable (PRECISE 3-NonV), and no csPCa was found. In the PRECISE 3-V group, csPCa was detected in 27.3% of patients.

**Table 2:** Repeat biopsy outcomes stratified by initial PI-RADSv2.1 and PRECISE scores

	PI-RADS 2	PI-RADS 3	PI-RADS 4–5	Total
PRECISE 1–2	0	13	6	19
ciPCa	0 (0)	1 (7.7)	1 (16.7)	2 (10.5)
csPCa	0 (0)	0 (0)	0 (0)	0 (0)
PRECISE 3-NonV	32	0	0	32
ciPCa	2 (6.2)	0 (0)	0 (0)	2 (6.2)
csPCa	0 (0)	0 (0)	0 (0)	0 (0)
PRECISE 3-V	0	6	5	11
ciPCa	0 (0)	1 (16.7)	0 (0)	1 (9.1)
csPCa	0 (0)	1 (16.7)	2 (40.0)	3 (27.3)
PRECISE 4–5	28	7	7	42
ciPCa	5 (17.9)	0 (0)	0 (0)	5 (11.9)
csPCa	10 (35.7)	5 (71.4)	7 (100.0)	22 (52.4)
Total	60	26	18	104
ciPCa	7 (11.7)	2 (7.7)	1 (5.6)	10 (9.6)
csPCa	10 (16.7)	6 (23.1)	9 (50.0)	25 (24.0)

*PI-RADSv2.1* Prostate Imaging Reporting and Data System version 2.1, *PRECISE* Prostate Cancer Radiological Estimation of Change in Sequential Evaluation, *ciPCa* clinically insignificant prostate cancer, *csPCa* clinically significant prostate cancer

**Fig. 4:**
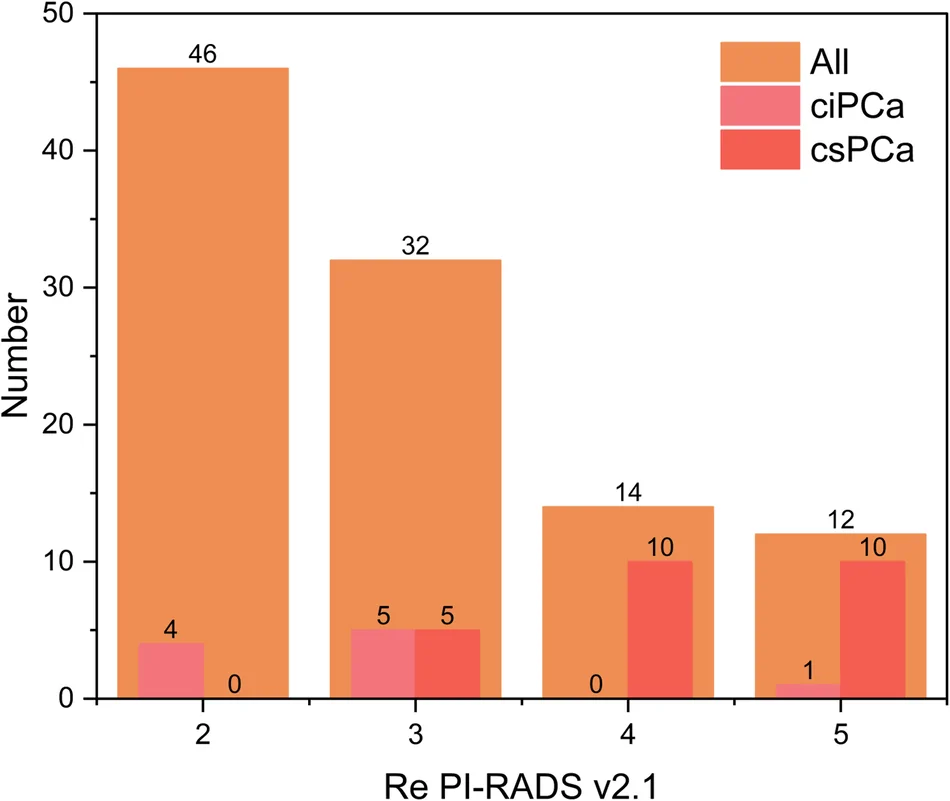
Distribution of ciPCa and csPCa in populations with different Re-PI-RADSv2.1. ciPCa, clinically insignificant prostate cancer; csPCa, clinically significant prostate cancer; Re-PI-RADSv2.1, Prostate Imaging Reporting and Data System version 2.1 score on repeat MRI

In addition, we summarized cancer detection rates by re-PI-RADS category (Fig. 4). CiPCa was detected in only 4 of 46 patients (8.7%) with re-PI-RADS 2. In contrast, csPCa was detected in the majority of patients with re-PI-RADS 4 (10, 71.4%) and 5 (10, 83.3%), with one case of ciPCa detected in re-PI-RADS 5. Among the re-PI-RADS 3 patients, five csPCa and five ciPCa were detected.

### Subgroup analysis

**Fig. 5:**
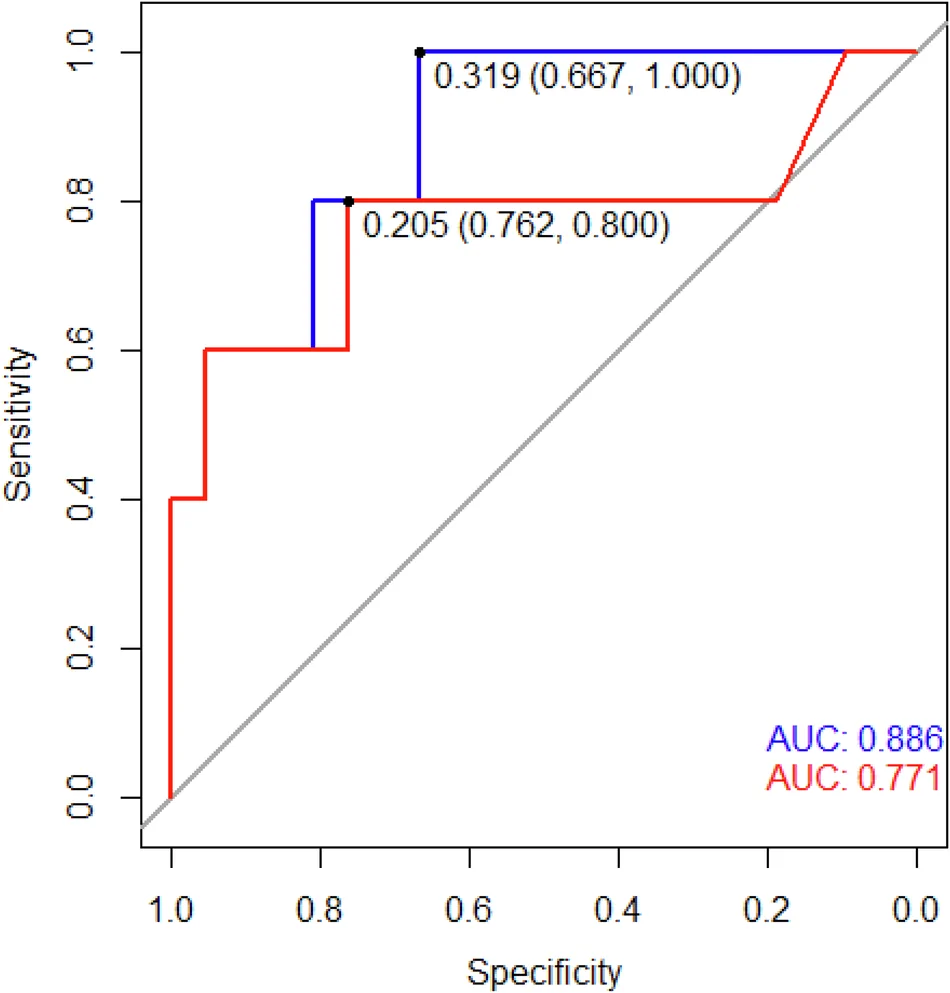
ROC curve analysis for the performance of Re-PSAD (red line) and PSAV (blue line) for men with Re-PI-RADSv2.1 3 and PRECISE 3–5

We performed a subgroup analysis focusing on 26 patients who progressed to a re-PI-RADS score of 3 or maintained this rating in subsequent MRIs. For these patients, it was challenging to ascertain whether a repeat biopsy was necessary based solely on MRI findings. To explore the predictive effects of other variables on csPCa, ROC curves are shown in Fig. 5, and the relevant parameters are shown in Table 3. The re-PSAD for csPCa prediction had an AUC of 0.771 (0.446–1.000). Using a threshold of 0.205 ng/mL^2^ for re-PSAD would have avoided 65.4% of repeat biopsies, but 5.9% of csPCa cases would have been missed. The AUC for PSAV was 0.886 (0.736–1.000), with a cutoff value of 0.319 ng/mL/year. If this were used as a criterion to assess whether a patient needed a repeat biopsy, no csPCa cases would have been missed in this study, and 53.8% of biopsies could have been avoided.

**Table 3:** Subgroup analysis

	AUC (95% CI)	Cutoff value	Sensitivity	Specificity	Biopsies avoided	CsPCa missed
Re-PSAD (ng/mL^2^)	0.771 (0.446–1.000)	0.205	0.800	0.762	65.4%	5.9%
PSAV (ng/mL/year)	0.886 (0.736–1.000)	0.319	1.000	0.667	53.8%	0.0%

*Re-PSAD* prostate-specific antigen density before repeat biopsy, *PSAV* prostate-specific antigen velocity, *AUC* area under the curve, *CI* confidence interval, *csPCa* clinically significant prostate cancer

## Discussion

In this cohort of men with persistent suspicion of PCa after a negative initial biopsy, csPCa was detected in 24% at repeat biopsy, largely among those with MRI-visible lesions on repeat imaging. The principal finding is that PRECISE scores strongly correlate with pathological outcomes in the repeat biopsy setting: csPCa was detected in 52.4% of patients with radiological progression (PRECISE 4–5), whereas no csPCa was identified in men with radiological regression (PRECISE 1–2). These results suggest that applying PRECISE to sequential MRI may help refine indications for repeat biopsy.

The absence of csPCa in the PRECISE 1–2 group (0/19) represents a crucial opportunity to reduce over-biopsy. This finding corroborates recent evidence regarding the natural history of MRI lesions. For instance, Becher et al prospectively studied men with PI-RADS scores of 4 or 5 who had no evidence of csPCa in prior biopsies [20]. The study suggests that only men demonstrating a downgrade to PI-RADS 2 can safely avoid repeat biopsy. Similarly, in the AS setting, radiological regression is strongly associated with prolonged upgrading-free survival [15, 19]. Our data extend these insights to the post-negative biopsy population, supporting a strategy where repeat biopsy may be deferred for patients showing radiological regression, provided that continued monitoring is maintained.

Conversely, radiological progression (PRECISE 4–5) was highly predictive of malignancy, supporting prompt repeat biopsy. In our cohort, the detection rate reached 100% for patients with both an initial PI-RADS 4–5 and a PRECISE score of 4–5. This aligns with the study by Meng et al, which reported that 62.5% of men with persistent PI-RADS 4/5 abnormalities on serial MRI were found to have cancer upon repeat biopsy [21]. Furthermore, Caglic et al demonstrated that PRECISE scores of 4–5 yielded a high sensitivity for predicting histological progression in AS cohorts [19]. Our findings suggest that “radiological progression” implies a high probability of a false-negative initial biopsy or rapid tumor growth, necessitating aggressive re-evaluation rather than continued observation.

A major challenge remains in managing patients with radiologically stable findings (PRECISE 3). Our data revealed that within the PRECISE 3 cohort, the presence of a visible focal lesion on MRI (3-V) was associated with a higher csPCa detection rate compared to nonvisible lesions (3-NonV), especially in patients with initial PI-RADS 4/5. Consistent with the conclusions of Stavrinides et al, any persistent lesions require close monitoring [22]. We also discovered that for patients maintaining or progressing to an equivocal score (re-PI-RADS 3), relying solely on imaging was insufficient to accurately identify those needing a repeat biopsy. Given this uncertainty, we introduced re-PSAD and PSAV to improve risk stratification for this subset. Our ROC analysis identified a re-PSAD cutoff of 0.205 ng/mL² and a PSAV cutoff of 0.319 ng/mL/year. The PSAD threshold is slightly higher than the conventional 0.15 ng/mL² recommendation [23], and the PSAV threshold is also notably higher than the commonly referenced 0.27 ng/mL/year [24]. Another important consideration is that while the PSAV cutoff value achieves perfect sensitivity (1.000), its specificity remains relatively low (0.667), potentially leading to more false-positive biopsy recommendations and unnecessary repeat biopsies. We believe these discrepancies are largely attributable to the study’s limited sample size, which may introduce significant variability. Although deriving a cutoff value based on the highest Youden’s index is methodologically sound, the generalizability of our findings is constrained by the small cohort and single-center design. The primary aim of this analysis was to offer a practical approach for patients with indeterminate imaging results, leveraging PSAV or PSAD, both established predictors of csPCa, to guide decisions on repeat biopsy. However, more precise cutoff values should be validated through prospective, large-scale studies.

This study has several limitations. First, due to the lack of standardized patient follow-up and timing of repeat biopsies, our results may be biased toward certain participants, despite indications being in line with current international guidelines. Second, although the study covers a period of approximately a decade, the sample size is relatively small, which may introduce statistical variability into the data, particularly in the calculation of the re-PSAD and PSAV cutoff values. Furthermore, as this was a small, single-center retrospective study, the conclusions drawn may have limited generalizability. Additionally, the long study period may have resulted in variations in the quality of the MRIs obtained, potentially affecting the assessment of imaging changes.

In light of these limitations, further research with a larger and more diverse sample size, as well as more standardized protocols, is needed to validate our findings and enhance their generalizability.

## Conclusions

In this cohort, PRECISE was associated with csPCa detection at repeat biopsy and may serve as a useful adjunct for assessing the need for repeat biopsy after an initial negative result. Combined with PI-RADS and PSA-based parameters, it may improve risk stratification, particularly in men with equivocal repeat MRI findings.

## Data Availability

Data are available upon reasonable request from the authors.

## Abbreviations

AS: Active surveillance
AUC: Area under the curve
bpMRI: Biparametric magnetic resonance imaging
ciPCa: Clinically insignificant prostate cancer
csPCa: Clinically significant prostate cancer
DRE: Digital rectal examination
IQR: Interquartile range
mpMRI: Multiparametric magnetic resonance imaging
PCa: Prostate cancer
PI-RADS: Prostate Imaging Reporting and Data System
PRECISE: Prostate Cancer Radiological Estimation of Change in Sequential Evaluation
PSAD: Prostate-specific antigen density
PSAV: Prostate-specific antigen velocity
ROC: Receiver operating characteristic
tPSA: Total prostate-specific antigen
TRUS: Transrectal ultrasonography
